# Supplementary material for: Functional networks of inhibitory neurons orchestrate synchrony in the hippocampus
Source: PLoS Biol. 2024 Oct 14;22(10):e3002837. doi: 10.1371/journal.pbio.3002837 (PMC11501041; doi:10.1371/journal.pbio.3002837)
Supplement: S1 Table — (DOCX) [file pbio.3002837.s008.docx]

|  |  |  |  | **Direct response** | **Indirect responses** | |
| --- | --- | --- | --- | --- | --- | --- |
| **Mouse ID** | **n FOVs** | **n stimulated interneurons** | **Total n Int** | **n stimulation trials with response** | **Total n positively modulated neurons** | **Total n negatively modulated neurons** |
| **ID_1** | 11 | 39 | 267 | 119 | 22 | 4 |
| **ID_2** | 9 | 32 | 193 | 61 | 2 | 3 |
| **ID_3** | 6 | 22 | 126 | 74 | 1 | 4 |
| **ID_4** | 8 | 18 | 107 | 101 | 4 | 11 |
| **ID_5** | 8 | 15 | 76 | 21 | 1 | 0 |
| **ID_6** | 4 | 10 | 47 | 41 | 9 | 1 |
| **ID_7** | 2 | 4 | 17 | 7 | 0 | 0 |
| **ID_8** | 1 | 3 | 26 | 4 | 0 | 0 |
| **ID_9** | 2 | 3 | 10 | 1 | 0 | 0 |
| **ID_10** | 1 | 2 | 6 | 3 | 0 | 0 |
| **ID_11** | 1 | 1 | 4 | 1 | 0 | 0 |
