# Supplementary material for: Functional networks of inhibitory neurons orchestrate synchrony in the hippocampus
Source: PLoS Biol. 2024 Oct 14;22(10):e3002837. doi: 10.1371/journal.pbio.3002837 (PMC11501041; doi:10.1371/journal.pbio.3002837)
Supplement: Attachment — Submitted filename: Response to reviewers.pdf [file pbio.3002837.s020.pdf]

We would like to thank all of the reviewers for their constructive evaluation, their excellent attention to detail, and their many helpful suggestions which we believe have significantly improved the quality of our manuscript. We are pleased that all reviewers appreciated the significance and importance of our work: Reviewer 1 acknowledged, "results are interesting, and the all-optical approach has its advantages," while Reviewer 2 highlighted "elegant manipulations with a very attractive technology" and the importance of the explored questions. Reviewer 3 commended the study as "technically and conceptually innovative." The concerns were mainly technical for the labelling method of inhibitory neurons (reviewer 1), regarding re-analysis of the data (reviewer 2) or about the citation and discussion of the past literature (reviewer 3). This revised version of the manuscript addresses these concerns comprehensively. In particular: (1) we provide in depth characterization of the mouse line and viruses used to control for unspecific expression of tdTomato or the opsin, which is the only concern shared by multiple reviewers; (2) we provide additional immunohistochemical analyses to demonstrate the specificity of our approach and identify the neuron types that were labelled in our GAD67-Cre line; (3) we provide additional analyses/modelling and statistical tests; (4) the introduction and discussion were updated following reviewer#3 suggestions, to improve their scholarship.

Reviewer #1 (Remarks to the Author):

The activity of inhibitory interneurons is critical for cortical functions, ranging from precise inhibition of excitatory neurons to coding memory information. Bocchio et al. used an all-optical approach to simultaneously record hippocampal interneurons and pyramidal cells during awake immobility. They reported that interneurons can promote synchrony through disinhibition without changing endogenous cell assemblies. The results are interesting, and the all-optical approach has its advantages. However, I have several concerns, especially regarding the methods they used to define interneurons.

1: Inhibitory interneurons are very diverse. In CA1, different types of interneurons are known to function distinctly. It is risky and imprecise to use GAD67 to mark inhibitory neurons, as it includes different types of inhibitory neurons and may likely affect the conclusions the authors are trying to make.

While we acknowledge that CA1 interneurons are diverse in structure and function and can be classified into different types, virtually no study to date has directly probed the action of single interneurons on multi-neuron activity. We believe that examining first all interneurons regardless of their class is a useful strategy. First, it does not introduce biases or exclusions or rare cell types. Second, it allows us to sample from a larger sample of interneurons, which arguably confers stronger validity when looking at correlations. Consequently, a deliberate choice was made to employ this particular strain of mice, facilitating the selection of interneurons from the broadest possible range to ensure comprehensive sampling. This strategy enabled us to discover that despite the spectrum of interneurons (over 30 subtypes identified in CA1 so far), the activation of single interneurons led to relatively homogeneous effects. One of the greatest sources of variability appeared to be the strength of activation of the targeted interneurons. Finally, despite our broad targeting approach, the fact that we imaged only in the stratum pyramidale allows to restrict our findings to fewer interneuron populations (basket cells, axo-axonic cells, two types of interneuron-selective interneurons and some bistratified cells). We appreciate the reviewer's suggestion to provide additional details on our experimental strategy, mouse line validation, and the specific interneuron types targeted. We have incorporated this information into the Methods, Results and Discussion sections to provide a more comprehensive understanding of our approach and its implications.

2: As mentioned above, expressing ST-ChroME in GAD67 neurons and optogenetically stimulating them does not make sense, as the authors have no information to identify what they are stimulating. Are they PV

neurons or SST neurons, which target excitatory neurons or inhibitory neurons in different neuronal subregions with distinct functions?

We understand the reviewer's concern, but we disagree on the point that stimulating single interneurons without knowing their full identity "does not make sense". A vast body of knowledge on inhibitory circuits originates from studies in which inhibition was studied without breaking down interneurons into specific subpopulations (e.g. Pouille and Scanziani, Science, 2001; Csicsvari et al., J Neurosci, 1999; Dupret et al., Neuron, 2013; Bonifazi et al., Science, 2009; all >700 citations). Even more recent studies have employed broad approaches to reveal the dynamics of inhibition in vivo (e.g., English et al., Neuron, 2017; Najafi et al., Neuron, 2021). Similarly to what we have argued in the point above, dissecting the role of interneurons in hippocampal co-activity by starting with a broad approach seems a logical rationale.

Our study follows a similar rationale, aiming to dissect the role of interneurons in hippocampal co-activity through a broad approach. By avoiding restrictions based on specific genetic markers, we were able to maximise sampling, a critical strength of our study. Unlike studies employing single-unit recordings, which often sample only a few interneurons due to technical limitations, our approach allowed for comprehensive sampling across multiple interneuron populations within each recording.

Importantly, imaging PV and SST interneurons does not confer full specificity (e.g., imaging PV neurons would tag both perisomatic-targeting basket and axo-axonic cells, but also dendrite-targeting bistratified cells). We agree with the reviewer that future studies focusing on specific interneuron populations will undoubtedly provide valuable insights. In response to this concern, we have provided additional information on the markers expressed by the neurons we imaged (Fig. S1, pages 2-3 and 16-17) and included speculations on the interneuron types contributing to the observed effects in the Discussion (page 11).

3: GAD67 can only mark a portion of inhibitory neurons, which does not allow authors to generalize their results to all inhibitory interneurons.

To the best of our knowledge, the GAD67-Cre line is an excellent line to tag most if not all GABAergic neurons in the hippocampus. GAD67 is expressed in virtually all MGE-derived and CGE-derived inhibitory neurons (quantified in Tricoire et al., J Neurosci, 2011). The GAD67-Cre line was initially carefully characterised in Tolu et al. the FASEB journal, 2010. Despite this strong previous evidence validating the use of GAD67 as a general marker for GABAergic interneurons, we have performed further validations (see Fig. S1 and page 17). Virtually all neurons expressing PV, calretinin and nNOS in the stratum pyramidale (the main interneuron subpopulations in this layer) were co-labeled by tdTomato expressed under the GAD67 promoter (Fig. S1d-f). Additionally, we found extensive co-localization between GAD67 and GABA (Fig. S1b). Finally, ST-ChroME expression under the GAD67 promoter showed a similar distribution to the known spatial pattern of GABAergic neurons in the hippocampus. Altogether, this evidence indicates that GAD67-Cre mice represent a suitable tool to study all GABAergic neurons in the hippocampus.

4: I am not sure whether the virus expressing in this paper is specific to inhibitory neurons or not. The authors should provide evidence to show their virus targeting is specific (not expressed in excitatory neurons) and the stimulation is really single-cell specific.

We have performed additional immunohistochemical analysis to address the first point (see page 17 and Fig. S1). We found that the vast majority of TdTomato neurons were immunopositive for GAD67 and/or GABA (Fig. S1a-c). In addition, the distribution of ST-ChroME expressing cells corresponds to the known distribution of GABA neurons in CA1 (Fig. S1g). In line with this, immunolabeling showed that ST-ChroME expressing cells were GAD67 positive (Fig.S1i-l).

Our optical stimulation of single neurons has already been characterised and validated in GABAergic neurons of the barrel cortex (Bollmann et al., Nature Neuroscience, 2023). The small lateral size of the excitation spot (~15 μm) and the use of a soma-targeted opsin (ST-ChroME), reduces the chance of photostimulating the neurites of non-targeted neurons. Additionally, GABAergic neurons are sparse and scattered, thus the chances of having two nearby neurons expressing the opsin are extremely low. Altogether, this approach leads to extremely low chances of stimulating more than one neuron at once. We have added this information to the Methods (page 18).

This is corroborated by the finding that the distance from the targeted neuron did not differ between positively modulated neurons, negatively modulated neurons and unmodulated neurons (that Fig. S3a).

5: Calcium spikes in the soma may not reliably reflect neuronal activities. It is likely that many synaptic events occur, regulating cell assemblies, but these may be missed in the analysis here.

We agree that calcium fluorescence transients do not report subthreshold synaptic inputs. However, 'cell assemblies' are typically defined as consistently co-active neurons, a definition that only requires the measurement of the output (spikes) of the neurons. A widely accepted method to define cell assemblies is extracellular electrophysiological recordings, which are also unable to record synaptic events. The only approaches able to record synaptic events as well would be in vivo patch clamp recordings or voltage imaging, but at present these approaches allow recording only a few neurons at the same time. The strength of our study is the ability to record hundreds of pyramidal cells and ~10 interneurons (on average) at the same time, which is essential to calculate meaningful correlations, synchronous calcium events and cell assemblies.

We are conscious of the fact that calcium imaging may miss a portion of single spikes compared to electrophysiological approaches. We now acknowledge this more extensively in the Results (lines 116-118) and in the Discussion (lines 527-530 and 535-537). However, while electrophysiology confers a better temporal resolution, it implies significant drawbacks, namely poorer spatial resolution (especially for stimulation purposes), less decisive identification of inhibitory neurons (if only based on spike waveform and firing rate) and more limited numbers of interneurons recorded. Thus, considering all these aspects altogether, our all-optical approach represents a useful tool to dissect how inhibitory neurons regulate cell assemblies.

Minor note: In the methods, the units for their holographic stimulation were not displayed properly (a font issue)."

Thank you for noticing this issue. This has been corrected.

Reviewer #2 (Remarks to the Author):

In this report, the authors combined holographic stimulation and calcium imaging to describe the impact of single-interneuron activation in the CA1 network. Interestingly, they describe a disinhibitory effect, with a higher proportion of interneurons becoming suppressed in the 1-second windows following the single-interneurons manipulation and a higher fraction of pyramidal cells becoming positively modulated. Next, by simulating this single-interneuron perturbation in a network model, the authors conclude that a cell assembly circuit architecture and dense but unspecific connectivity between interneurons explain the

experimental results. Finally, the authors show that the transient increase in excitability upon disinhibition mediated by single-interneuron stimulation increases the probability of ripple-like population events.

These are elegant manipulations with a very attractive technology, and the questions explored are important and timely, but unfortunately, there are some important drawbacks in the present work that require careful consideration:

Major points:

- A critical analysis that is missing throughout the manuscript is controlling for the rate differences within groups or between pyramidal cells and interneurons. For instance, the authors find higher pairwise correlations between interneurons than correlations between pyramidal cells in Fig. 1f-g, but it is unclear how much of this result could be explained by the differences in the frequency of calcium fluorescence signals between groups (Fig. 1c). The authors should include a comparison of those neurons with similar ΔF/F in the interneuron and pyramidal cell groups and show if the difference still exists, or even better, correlate the ΔF/F against the average pairwise correlation for each neuron in both groups or downsample randomly (100 times or more) calcium transients so that ΔF/F is similar among cell types.

The same concern applies to the pyr-pyr to pyr-int correlations in Fig. 1h and the percentage of SCEs and pyr-int correlations in Fig. 1i3, etc. Could more active neurons show higher correlations than their counterparts with lower rates? Here, the authors could color-code the dots by correlating the average ΔF/F of the pyramidal cell to see if a pattern arises and/or quantify these effects with an ANCOVA. Similarly, rate effects should be disambiguated in Fig. 4a.

Thank you for your valuable feedback regarding the need to control for rate differences within groups and between pyramidal cells and interneurons. If the reviewer refers to a general concern related to firing rates affecting correlations (e.g. present also in electrophysiological data), correlations - unlike covariances - are intrinsically normalised to standard deviations, thus not affected by rate differences. If, however, the reviewer refers to an indirect technical bias (e.g. different baseline fluorescence leading to a difference in transient amplitudes), it is true that in the previous version of our manuscript we could not rule out that such indirect bias could affect our findings. To control for potential biases in the correlation results due to different ΔF/Fs between the two populations, we subsampled pyramidal cells to match interneurons' ΔF/Fs in each recording. In these conditions, interneurons still showed significantly higher pairwise correlations ($p=0.042$, $n=11$ FOVs from 6 mice, Wilcoxon signed rank test, Fig. S1c). This control is now described at page 3 (lines 128-133). Thus, higher rates of interneurons do not seem to affect calcium dynamics in a way that biases pairwise correlations.

- The results on the circuit responses to single-interneuron photoactivation (Fig. 2) and the network model are very interesting (Fig. 3), but it is not clear to me how much the model results depend on the chosen parameters, and statistical quantification of the results is missing. Besides, if the main point of the authors is that the absence of parcellation of inhibition in the model explains the experimental results, I believe that the authors should keep the probability of connection constant between I and I (as they did in Extended Data Fig. 3a instead of Fig. 3c).

Many thanks for raising these important points. (A) First, we performed further simulations to show that our results are robust to the choice of parameters. (B) Second, we quantified our results by performing statistical tests and showed that the observed changes are significant.

(A) New simulations showed that the results of our single inhibitory neuron perturbations are robust to the choice of network parameters (Fig. S6). We simulated our network models with different ranges

of parameters and calculated the modulations in each network. Specifically, we tested the dependence of our results on key parameters: the coupling strength in the network (J), and the connection probability of E-E and E-I connections. Increasing or decreasing J by a factor of two did not change our results (Fig. S6b). Similarly, increasing the connection probability of sparse E-E connections (from 1% to 10%), and decreasing the connection probability of E-I connections (from 50% to 25%), led to similar results (Fig. S6c,d). We therefore conclude that our results are robust to the choice of parameters in the network as our results hold for a wide range of parameter space.

(B) We quantified whether our main results, regarding the response changes as a result of single neuron inhibition, were statistically significant (Fig. S5). We calculated the fractions of excitatory (E) or inhibitory (I) neurons that are positively or negatively modulated, respectively, upon perturbing each single I neuron in the model. We then ran a paired t-test to quantify if the distribution of positively and negatively fractions are statistically different. Differential modulation of E and I neurons were statistically significant in the default model (Fig. S5a). Removing the subnetwork structure kept the pattern of modulations in I neurons, but abolished the pattern in E neurons, which expressed a non-significant difference between their positively or negatively modulated fractions (Fig. S5b). The reported changes as a result of less global I-I and denser, specific I-I connectivity were also both significant (Fig. S5c,d).

Regarding the connection probability of I-I connections: the main insight obtained from our modelling result was that both specific E-I subnetwork structure *and* global I-I connectivity are necessary to explain the experimental results. The “global” condition generally refers to both the density (higher connection probability) and nonspecificity (lower connection specificity) of I-I connections. In Fig. 3c we show that, when both conditions are violated (C1: I-I connection density is reduced, from 85% to 50%, to become comparable to E-I connections *and* C2: I-I connections are as specific as E-E and E-I connections), the results deviate from the experimental findings. To further scrutinise these conditions separately, in the supplementary figure (Fig. S5d, in the updated figures), we consider the situation where only C2 is violated (namely, I-I connections are specific but still dense). This combination revealed that, even when connections are specific, dense I-I connections can lead to qualitatively similar results as experimental findings, although to a weaker degree than the default network (compare Fig. S5d and Fig. S5a). This suggests that the joint violation of C1 (density) and C2 (nonspecificity) is needed to abolish our results.
To summarise, in Fig. 3a both C1 and C2 are holding. In Fig. 3c, both C1 and C2 are violated. In Fig. S5d, C1 is holding but C2 is violated.

- In my opinion, the results in Fig. 4a are not strong enough to support the claims of the authors. I believe that it is not surprising that the assembly activation-triggered average of a high number of pyramidal cells unrelated to one interneuron shows no effect when that interneuron is firing or not, considering that not even those pyramidal cells belonging to the same assembly showed any effect. I wonder if the authors could look at nearby interneurons or restrict the analysis to nearby high-rate pyramidal cells…

Thank you for your insightful feedback regarding the results presented in Fig. 4a. We acknowledge the limitations inherent in our analysis and understand your concerns. Specifically, we recognize that averaging the activity of a large number of pyramidal cells that are not directly related to a single interneuron might dilute potential effects, especially given that even pyramidal cells within the same assembly did not show significant effects.
The suggestion to examine nearby interneurons or restrict the analysis to nearby high-rate pyramidal cells is indeed valuable. However, due to the sparse distribution of interneurons and their typically low numbers within assemblies, implementing such analyses poses significant challenges. Moreover, defining "high-rate" pyramidal cells in the context of calcium imaging is complex and could introduce arbitrary thresholds. High

rate could refer to the frequency of calcium transients, high ΔF/F, or even high baseline fluorescence, each of which could be interpreted differently.
Given these technical constraints, we agree that our current analysis may not be fully conclusive. To address this, we have relocated the panel to the Supporting Information section (now Fig. S7d) and have expanded the discussion of these limitations in the main text (pages 11-12).

Minor points:

- ‘Orthogonal’ (line 60) usually describes events that are statistically independent or do not affect one another in terms of outcome, not mutually exclusive or competing events.

We have substituted the term "orthogonal" with "competing," as per your suggestion.

- What does it mean that the interneurons and pyramidal cells are recruited in the same proportion during SCEs? When looking at extracellular spikes, typically a higher proportion of interneurons fire during ripples as compared to pyramidal cells (Csicsvari et al., 2000).

The difference between our observations and previous ones using electrophysiological recordings such as Csicsvari et al. 2000 can be explained by the fact that the identification of interneurons through the clustering of extracellular spikes is biased towards detecting fast spiking PV-basket cells which is known as the interneuron subpopulation the most recruited during ripples (together with TORO cells cf. Szabo et al. 2022). We have added a sentence in the discussion to explain this point (see lines 527-530).

- In Line 191, I believe that the authors want to say Fig. 2b-c instead of Fig. 3b-c.

Corrected, thanks.

- How do authors justify statistically calling relatively similar proportions to 68% and 32% (line 211)?

We thank the reviewer for spotting this imprecision. We have corrected this sentence.

Reviewer #3 (Remarks to the Author):

The present paper by Bocchio et al. uses an elegant experimental approach to probe the role of individual inhibitory interneurons (INs) in shaping pyramidal neuron network activity in hippocampal area CA1. The authors report that CA1 INs form a functionally interconnected network to exert synchrony-promoting effects on pyramidal neurons (PNs), presumably via disinhibition. While the study is technically and conceptually innovative, major concerns regarding scholarship, interpretation, presentation, and therefore impact significantly undermine confidence in this manuscript in its currently-written form.

Major Concerns:

1. The finding that CA1 interneurons promote synchrony is presented as surprising and paradoxical. This framing would require a rather selective sampling of the hippocampal literature. A significant number of

studies (in fact too many to reasonably enumerate here) have demonstrated a role for inhibition in promoting synchrony among networks of pyramidal neurons in various cortical regions, including the hippocampus (e.g. SWRs, which are prominently discussed in the manuscript). Many such studies point to a role for perisomatically-targeting INs, which presumably comprise the bulk of the INs in this study given imaging was carried out exclusively in stratum pyramidale. Rather than omitting this body of work in favor of amplifying the surprising nature of the study's findings, a balanced portrayal of past literature would improve the impact of this study by facilitating its integration into the body of existing knowledge regarding how INs shape PN assemblies that ultimately comprise CA1 output.

We apologise for this oversight. Our intention was not to omit this literature. The introduction and discussion have been thoroughly re-written to take all previous work into account.

2. Another bold and poorly cited assertion, which serves to build to premise for the present study, is that CA1 PNs largely rely on intrinsic rather than external inputs. It is unclear whether "external" indicates inputs originating outside CA1 or outside the entire hippocampal formation. If the former, this statement would seem fairly outlandish and is heavily contradicted by one of two provided references. Both entorhinal and CA3 inputs to CA1 are feature-tuned at the level of individual neurons with compartment-specific projections and, in the case of CA3, clearly defined sub-dendritic synaptic clustering (Adoff, et al 2021, Nat. Comm.; O'Hare, et al 2023, Science) which is widely deemed critical in driving local dendritic computations giving rise to feature selectivity (see numerous studies by Karen Zito, Kristen Harris, Yiota Poirazi, and Bartlett Mel), a major determinant of CA1 PN dynamics. Additionally, Terada et al (2023, Nature) demonstrated that CA3 inputs terminating in CA1 stratum radiatum organize to form compressed replay sequences around "HSEs" (analogous to the authors' favored SCE acronym) that tend to occur with SWRs. How then can SWR-associated CA1 sequences be entirely driven by local feedforward and feedback inhibition? The myopic acknowledgement of existing literature, in a manner that appears tailored to the study's aims and findings, is unfortunate. Moreover, it is again unnecessary: the role of INs in shaping orthogonalized PN assemblies remains understudied and this manuscript makes a valuable contribution in that regard.

We appreciate the reviewer's detailed feedback and acknowledge that our initial manuscript presented an overly narrow interpretation of the role of interneurons. We now include a paragraph of the discussion that mentions many of the studies suggested by the reviewer, proposing that the influence of interneurons acts on top of this layer of mechanisms involving excitatory inputs and synaptic clustering onto dendrites (see pages 10-11).

3. Final paragraph of Introduction: "We show that CA1 interneurons favor the co-activity of pyramidal cells in the form of pairwise correlations and Sharp Wave Ripples (SWRs)-associated network bursts"

• This sentence is too terse to parse, particularly for a non-neuroscience-specific journal. Pairwise correlations between what – PN-PN, IN-PN, both? What exactly is being implied about SWR-associated synchronous events?

We thank the reviewer for spotting this unclear sentence. We have now re-written it.

• SWRs were not measured. The Cossart lab has previously established that SCEs tend to (but do not always) occur during SWRs (again, as was also shown in CA3, a primary source of excitatory input to CA1 PNs and IN, by Terada et al which is not cited). Of course a new study can and should be linked to past work, but this must be done responsibly. The authors do not know, in their current experimental conditions, how many and exactly which SCEs were associated with SWRs or when/how often SWRs even occurred in their recordings.

We agree with the reviewer. We have toned down our reference to SWRs throughout the text (introduction and discussion). We now cite the Terada et al. 2022 paper at page 10 (lines 477-479).

4. Even though mentioned briefly in the discussion, it should be pointed out clearly in the introduction and results that this study is solely based on interneurons that reside in stratum pyramidale and thus mostly on perisomatic targeting interneurons. Indeed, two of the cells in Fig 1b do not show increased activity during locomotion (if anything, they seem to increase their activity at the cessation of locomotion) and yet the corresponding text in the legend states : “Note the increased activity in most cells during locomotion and larger calcium transients in interneurons.“ On what timescale was locomotion modulation assessed? Would finer-timescale events associated with the onset and offset of locomotion epochs have been washed out? Was IN activity regressed against acceleration, which would be more likely to catch these phenomena?

We appreciate the reviewer's insights and recognize that our mention of locomotion modulation in the figure legend may have caused some confusion. In this study, we did not specifically assess the modulation of interneurons by locomotion. Locomotion was used merely to define SCEs during rest periods and for a few control analyses. We acknowledge that examining the modulation of interneurons by locomotion and the diversity of their responses would be valuable. However, we believe that this would extend beyond the scope of the current paper and thus propose to address these analyses in future dedicated studies. To avoid further confusion, we have removed the misleading sentence from the legend of Fig. 1.

We have also clarified in the introduction and results that this study focuses exclusively on interneurons residing in the stratum pyramidale, primarily targeting perisomatic regions (e.g. lines 489-494, 498-501, 546-548). This specificity is crucial to accurately convey the study's focus and findings. Thank you for bringing this to our attention.

5. Related to concern (4): interneuron-selective cells are discussed (p. 9) as a potential source of disinhibition that may partially explain an inferred (!) disinhibitory effect; however, interneuron-selective cells primarily reside outside of stratum pyramidale. Please delineate precise inhibitory microcircuits, demonstrated in the CA1 literature, through which INs in pyramidale may largely inhibit one another. Based on that existing body of knowledge, what cellular/dendritic compartments are disinhibited and how ought this to inform the impact of disinhibition on PN somatic activity? For instance, distal dendrite-targeting INs may serve more to gate tuft-initiated synaptic plasticity than to orthogonalize cell assemblies. More broadly, it is surprising that not a single IN subtype is mentioned in the manuscript given the immense variety of in vivo firing patterns and cellular/subcellular targeting motifs that have been thoroughly documented in CA1. It is useful to image INs in bulk, but to then argue against the importance of this vast body of knowledge conveys motivated reasoning.

We thank the reviewer for providing this comment that significantly improves our manuscript. We now include a thorough discussion of potential interneuron types involved as well as putative mechanism (lines 492-511)

6. Certain interneurons are known to be connected via gap junctions. Authors should address this, e.g. by calculating the distance-dependence of IN-IN coupling during single-IN photostimulation experiments. Additionally, it should be acknowledged that the enrichment of gap junction-coupled INs in pyramidale would surely bias the study’s conclusions regarding IN-IN interactions.

We thank the reviewer for raising this valuable point. We agree that gap junctions could be common between interneurons, particularly between PV interneurons of the stratum pyramidale (Baude et al. 2007). These cells are coupled by cx36. Interestingly, SWRs were still observed in cx36 knock-out mice (Buhl et

al. 2003), which would suggest that gap-junctions among PV interneurons are not essential for promoting this type of synchronous events.

Additionally, the average distance between the stimulated interneurons and the indirectly modulated interneurons was 137 ± 52 um (6 neurons). Based on previous work, the frequency of coupling among fast-spiking interneurons in cortical regions is ~65% when the somata are less than 80 μm apart (Galarreta & Hestrin, Nature, 1999). In dual recording experiments from pairs of fast spiking interneurons separated by larger distances, the frequency of coupling was found to be much lower (~1%).

Altogether, these points suggest that gap junctions could play a role, but arguably a minor one compared to synaptic transmission. We now discuss this (lines 457-463).

7. P. 4: “We found that the activity of a small subset of imaged cells (62 neurons from 23 FOVs in 6 mice) could be identified as positively or negatively modulated by the stimulation of single interneurons (Fig. 3b-c).”

a. This does not inform as statistical analysis. The reader does not know (a) how those 62 neurons were distributed across FoVs and mice, how many neurons were in each FoV (i.e. the denominator), and therefore the FoV-to-FoV and mouse-to-mouse variability of percentages. That variability must be represented with error bars and statistical tests for significance.

b. Please include a table accounting for the number of cells/FoV and FoVs/mouse, with clarification as to how 23 distinct FoVs were acquired from only 6 mice with unilateral window implants.

Thank you for your detailed feedback regarding the description of our imaging data and the need for statistical analysis. We appreciate your suggestions for improvement and have made the following revisions:

a. We have included a table in the Supporting Information that provides a comprehensive breakdown of the number of cells per field of view (FoV) and the number of FoVs per mouse. This table allows readers to understand the distribution of imaged cells across FoVs and mice, providing context for the analysis of variability and significance. The 23 FoVs from 6 mice include only the FoVs in which indirectly modulated cells could be found.

b. Regarding the acquisition of 23 distinct FoVs from only 6 mice with unilateral window implants, we would like to clarify that the diameter of the cannula (3mm) exceeds the typical size of a single FoV (280 um * 280 um). Therefore, different FoVs can be sampled from a single craniotomy, enabling the imaging of multiple CA1 regions within the same mouse. This approach allows for efficient data collection while minimizing the number of animals used in the study.

Thank you for highlighting these important points. We believe that these revisions enhance the clarity and transparency of our methods and results.

8. Throughout the manuscript, statistics are confusing and the associated narrative seem to push a one-sided storyline. This takes the form of either omitting statistics altogether, or else referencing a difference in averages as notable when it is not significant. One example of each:

a. The entirety of Figure 3 lacks statistical tests for significance, despite every data summary figure being easily suitable for one; either through simple t-test/Mann-Whitney or else bootstrapping to calculate significance threshold.

Thanks for raising this point. We now quantified our results to see if the changes are statistically significant (Fig. S5). To do this, we calculated the fractions of excitatory (E) or inhibitory (I) neurons that are positively or negatively modulated, respectively, upon perturbing each single I neuron in the model networks. We then ran a paired t-test to quantify if the distribution of positively and negatively fractions are statistically different. Differential modulation of E and I neurons were statistically significant in the default model (Fig. S5a). Removing the subnetwork structure kept the pattern of modulations in I neurons, but abolished the pattern in E neurons, which expressed a non-significant difference between their positively or negatively modulated fractions (Fig. S5b). The reported changes as a result of less global I-I and denser, specific I-I connectivity were also both significant (Fig. S5c,d).

b. P. 7: “On average, the calcium transients of the same assembly were reduced in amplitude when the interneuron was inactive, but this effect was not statistically significant.” – The p-value here was not 0.07, which one might arguably call a “trend”, but 0.7.

We thank the reviewer for spotting this typo. This has now been corrected.

9. Please validate the usage of the GAD67-Cre line, i.e. whether GAD67 is expressed in all GABAergic cells residing within CA1 or, if not, which inhibitory IN subtypes.

A full characterization of the mouse line is now provided (Fig. S1). This mouse line allows for a large sampling from diverse interneuron subtypes in the CA1 pyramidal layer, including the most representative ones, the PV-expressing basket and bistratified cells, the NOS-expressing ivy cells and two types of interneuron-selective interneurons (ISI 1 and 3) that express calretinin (see Fig. S1 and Methods for quantification).

10. From The Jackson Laboratory’s description of the Ai14 mouse line used to Cre-dependently express tdTomato in GAD67(+) neurons: “Importantly, both Ai14 and Ai9 may exhibit low levels of tdTomato expression prior to exposure to Cre recombinase - but the tdTomato expression levels after Cre recombination are greater than those baseline levels. As such, it is recommended that researchers include Cre-negative controls to establish the baseline tdTomato levels in their experiments. See Detailed Description for additional details.”

a. Please validate IN targeting and, most importantly, classification based on fluorescence.

b. This point is of particular concern given the suspicious level of similarity between PN and IN GCaMP6m traces in Fig. 2b. IN Ca2+ dynamics are so slow and convolved (by Ca2+ buffering proteins) that most investigators eschew transient detection altogether, favoring dF/F. In fact, the typically higher baseline fluorescence in INs would predict the opposite of the larger IN transient amplitudes reported (again, with no statistical analysis/evidence) in Fig2b. A supplemental figure with a more comprehensive sampling of IN traces may be helpful.

We thank the reviewer for raising this point. We agree that nonspecific expression is often a matter of concern, particularly regarding reporter lines. We now include a full characterization of the specificity of tdTomato and ST-Chrome expression in GABAergic neurons (pages 16-17 and Fig. S1). The vast majority of Td-Tomato neurons (90.47%, n=5 animals, n=986 cells) were immunopositive for GAD67 and/or GABA (Fig. S1a-c). In addition, the distribution of ST-ChroME expressing cells corresponds to the known distribution of GABA neurons in CA1 (Fig. S1g). In line with this, immunolabeling showed that ST-ChroME expressing cells were GAD67 positive (Fig.S1i-l). We found no evidence of tdTomato or ST-Chrome expressing neurons with pyramidal-like structure (e.g. long apical dendrites reaching the lacunosum moleculare), whereas the expression of GCaMP6m in neurites across the CA1 layers is consistent with expression in the vast majority of pyramidal cells (Fig. S1g).

Regarding Ai14 mice, these account for only two out of six mice included in the imaging experiments with tdTomato (no optogenetic stimulation). We have previously characterized this line for previous projects and found virtually no tdTomato expression without Cre in the hippocampus. In this project, we have compared the proportion of recorded and segmented interneurons out of the total number of segmented neurons in the field of view between Ai14 mice (n=2) and mice in which tdTomato was expressed via viral means (n=4) and similar proportions (Ai14: mean 4.5%, range 3.7%-5.2%. Virus: mean 4.9%, range 3.4%-8.7%). Thus, our immunohistochemical analyses, the fact that the Ai14 approach did not label more interneurons than the viral approach, as well as the fact that the proportions of interneurons are no higher than the known proportion of interneurons in CA1 (10-20%), indicate that our approach did not lead to "leaky" expression of tdTomato in pyramidal cells. We have included the traces of all segmented interneurons from a representative recording in Fig. S2a). We would like to highlight that we have not used transient detection for most of the study. We typically quantified either baseline-normalised fluorescence or ΔF/F (see Methods). We only employed transient detection for detecting small events that are part of SCEs (as in Malvache et al., 2016).

11. If the main text referencing Fig 4c is indeed meant for that panel, it is difficult to parse. There appear to be cells on the contour plot that were recruited during stimulation and did not overlap with the recruited cells during baseline, which would contradict the authors' statement: "all of the pairs recruited in the same assembly during or just after stimulation were already part of a similar assembly during baseline". More generally, summary figures are insufficient to make any general claim. Dataset-wide data must be shown, with summary statistics and significance tests. Again, bootstrapping against a hypothesis is useful in the absence of an obvious standard test.

Thank you for your thorough review and insightful comments regarding Figure 4c. We appreciate your efforts to clarify the interpretation of this figure and address the concerns raised.

The main objective of Figure 4c is to illustrate that a simulation involving a single interneuron did not introduce new cells into previously identified cell assemblies, thus preserving the internal network structure. We acknowledge the challenge posed by the differing durations of the baseline period and the combined stimulation and post-stimulation periods, which may affect the identification of statistically significant assemblies. Consequently, some assemblies that were significant during the baseline period might not retain significance during the combined stimulation and post-stimulation period due to the shorter duration.

To clarify, our claim is not that the cells in assemblies for these two periods perfectly match. Rather, we focused on assessing the preservation of functional connections between pairs of cells considered part of any assembly in both periods. For example, if cell A and cell B are part of the same assembly during the stimulation period, we investigated whether they already belonged to the same assembly during the stimulation period.

In response to your feedback, we have revised Figure 4c to enhance clarity and performed additional analysis comparing SCE cell composition between baseline and stimulation periods (page 9 lines 382-402). This revision aims to provide a more intuitive representation of the data and facilitate clearer interpretation.

Thank you for bringing these concerns to our attention, and we hope the revised Figure 4c better addresses your queries and contributes to the overall clarity of our findings.

Minor:

1. Please insert line numbering through the manuscript.

Inserted.

2. Please correct several incorrect figure references. For example: Fig. 2b-c.

Corrected.

3. What do the circles represent on Figure 1c, d, g and i? What measures (e.g. quartiles) do the box charts show? Please include in the figure legend.

We have specified this information in all figures with boxplots.

4. Please correct typographical and grammatical errors. A subset of examples: p. 3, participated to > at; p. 45 minutes long > minute; p. 13: GCaMP6m, was injected; p. 28: bluet; p. 8: …, the fraction of them that already…

We thank the reviewer for spotting these typos. These have now been corrected.

5. Please reference figure panels in the order that they appear in figures (for example, Fig. 3a).

All figure panels are listed in order in the text.
