# Supplementary material for: Functional networks of inhibitory neurons orchestrate synchrony in the hippocampus
Source: PLoS Biol. 2024 Oct 14;22(10):e3002837. doi: 10.1371/journal.pbio.3002837 (PMC11501041; doi:10.1371/journal.pbio.3002837)
Supplement: Attachment — Submitted filename: R1_response to reviewer.docx [file pbio.3002837.s022.docx]

**Reviewer 1 (their own comment and their comments 2, 8, 11 on Reviewer 2's comments**

We thank the Reviewer for the very positive assessment of our revised manuscript.

Reviewer #1: In general, I believe that the manuscript has improved and that most of my comments have been addressed properly. A last minor point:

-	Low and high firing rate neurons can indeed bias pairwise correlations or other rate-derived metrics in both extracellular recordings and imaging data. Consequently, a decrease in statistical power, as found by the authors, is expected. However, I believe this is a relevant result, highlighting the specificity for interneurons in the analysis shown in Fig. 1c. I still think a similar analysis would be a desirable addition to some important panels for the narrative, such as Fig. 1i.

We have followed the Reviewer’s suggestion and employed the same subsampling approach for the findings related to Fig. 1e4 and Fig. 4a3. Indeed, we have found differences in DF/Fs between these conditions, but the subsampling approach revealed that higher correlation with interneurons for cells highly active in SCEs or participating to assemblies was maintained even when controlling for DF/Fs differences. We have included these findings in the Results main text and in Fig. S2f-g.

I believe that most of the comments from reviewer 3 have been either correctly addressed or thoroughly discussed.

-	Point 1. The new sections are largely different from the previous manuscripts and are now more clear and comprehensive.

-	Point 2. The point raised by Reviewer 3 is both valid and interesting, reflecting an ongoing debate in the field. It is well-established that CA1 neurons largely inherit their firing patterns from upstream CA3 and EC networks (Ahmed and Mehta, 2009; Brun et al., 2008; Franzius et al., 2007; Rolls et al., 2006; Savelli and Knierim, 2010; Solstad et al., 2006; Steffenach et al., 2005). However, recent experiments have shown that a significant fraction of CA1 pyramidal neurons remain place cells even in the absence of major EC and CA3 inputs (Zutshi et al, 2022 Neuron; in fact, this paper is specifically exploring this question: "Extrinsic control and intrinsic computation in the hippocampal CA1 circuit" ), and this results align with previous and current observations (Huszar et al., 2022, Nat Neurosci; Stark et al., 2013, Neuron). These findings suggest that the CA1 circuitry is intrinsically shaped by ontogeny (Farooq and Dragoi, 2019, Science; Huszar et al., 2022, Nat

Neurosci) and experience (Wilson and McNaughton, 1994, Science), enabling it to parse and describe the variance of inputs received from CA3 and EC as part of its spontaneous activity. The work of Bocchio et al. further investigates the inhibitory-excitatory synapses in CA1, which are major contributors to these intrinsic computations.

We believe that this point is resolved as the Reviewer was simply putting our findings in the context of other published studies.

-	Point 3: Terada et al. 2022 is cited, and SWR references are toned down.

-	Point 4. I concur with the reviewer comment about the fact that focusing on interneurons residing in the pyramidal layer restricts the experiment to mostly perisomatic targeting of interneurons. The two neurons increasing activity when locomotion stops can well be CCK-basket cells (Dudok et al., 2021 Neuron). I believe that the authors addressed this point correctly.

-	Point 5. Based on the overall results of the work, the new paragraph about interneuron types underlying local disinhibition is an important addition.

-	Point 6. In my opinion, and in light of the author's comment, the inclusion of gap-junction-mediated synchrony, which is potentially interesting, is out of the scope of this manuscript.

-	Point 7. The supplementary table certainly improves data interpretability and transparency and, in my opinion, addresses the reviewer concern.

-	Point 8. I believe that including some of the statistics of the network model (Fig. S5) as a main panel in figure 3 will strengthen the message of this figure.

We have now included the statistical comparisons as main panels in Fig. 3.

-	Point 9. Gad67 selectivity for GABergic types in CA1 is a major concern of reviewer 1 and has been extensively addressed by the authors.

-	Point10. Correctly addressed by the authors, in my view.

-	Point 11. In the current version, the authors have included in the text a statistical statement for Figure 4c: "All of the pairs recruited in the same assembly during or just after stimulation were already part of a similar assembly during baseline (median 100%, n = 236 pairs, 3 mice)," but I feel that a more comprehensive visual description of this important result (including statistics) will improve the manuscript.

We have performed a new analysis to better represent the findings and include statistics. Consistent with the observation on cell pairs, we have found that stimulation of single interneurons leads to activation of cell assemblies with a higher degree of overlap with previously expressed assemblies compared to chance. We have added this new analysis to the Results and Methods. The data are shown in Fig. 4c3
